# Effectiveness of GV20-based wearable low-level laser acupoint therapy for pain on head

**DOI:** 10.1007/s10103-026-04877-6

**Published:** 2026-05-07

**Authors:** Wen-Fang Lei, Arslan Sadiq, Zhen-Yang Cui, Xu-Bo Wu, Shin-Da Lee

**Affiliations:** 1 PhD Program for Health Science and Industry, China Medical University, Taichung, Taiwan; 2 Graduate Institute of Acupuncture Sciences, China Medical University, Taichung, Taiwan; 3 School of Rehabilitation Medicine, Shandong Second Medical University, Weifang, China; 4 Department of Rehabilitation Medicine, Pudong New District People’s Hospital, Shanghai, China; 5 PhD Program in Healthcare Science, College of Healthcare Science, China Medical University, Taichung, Taiwan

**Keywords:** Baihui, Headache, Laser Acupuncture, Pain, Visual Analog Scale

## Abstract

The effectiveness of GV20-based Wearable Low-level Laser Acupoint Therapy for pain on right temporal head was investigated. The study was designed as a triple-blinded, randomized controlled clinical trial. The deep pressure pain targeted by 300g monofilament and Visual Analog Scale (VAS, 0-10) scores on right temporal head were assessed were randomly assigned to wearable LLLAT (650 nm, 5 mW, 30 min, about 9 joule each acupoint) on Baihui (GV20) with four Sishencong (EX-HN1) or sham group (n=24 vs 24). The mean VAS pain scores in wearable LLLAT group after GV20 with EX-HN1 acupoint treatment were significantly decreased comparing with sham group. This trial suggested that wearable Low-level Laser Acupoint Therapy 650 nm 5 mW for 30 min each acupoint simultaneously could effectively reduce deep pressure pain on temporal head.

## Introduction

Head pain is one of the most prevalent neurological disorders and is a public health problem about 50–60% at least one head pain a year in all countries [1, 2]. Tension-type headache among the most common primary headache disorders about 38–42% of adults, characterized by recurrent episodes that are difficult to manage [3]. Migraine 12–15% of adults. Acupuncture, a well-recognized non-pharmacological treatment, is frequently employed for pain management. Acupuncture therapy has been applied to head pain around the world and were reported more than 800 research [4].

Non-invasive and needle-free Low-level Laser Acupoint therapy (LLLAT) has become more common used around the world due to perceived safety [5], especially for patients with needle phobias well as elderly people and children. LLLAT is well-developed around the world and a safer pain-free alternative to traditional acupuncture, with minimal adverse effects and greater versatility [5, 6]. LLLAT was reported to be a safer therapy than needle acupuncture [7]. However, the evidence about laser energy of LLLAT (wavelength, power, duration) in each acupoint is not uniformly high-quality.

GV20, also known as Baihui, is a crucial acupoint on the top of the head for treating head pain (deep pressure pain on head, tension-type headache or migraine), dizziness, insomnia, anxiety, and post-stroke-related neurological and cognitive functions [8]. The LI4 and GV20 are the two of most six frequently used acupoints to treat migraine head pain [9]. Without acupoint needling by an acupuncturist, the LI4 is the easiest self-manual acupoint LLLAT for treating pain. GV20 acupuncture has a beneficial effect on pain in patients with severe Fibromyalgia Syndrome [10]. Low-level Laser acupoint therapy (1.3 J each acupoint including LI4) was suggested an effective treatment for chronic tension-type headache [11] and can improve migraine or tension type headache in 9–15 years old children [12]. However, whether 30 min LLLAT on IL4 or GV20 can effectively relieve pain and how effectiveness of wearable GV20 LLLAT on head pain are still unclear.

To evaluate the effectiveness of wearable LLLAT of GV20 with four EX-HN1 on for deep pressure-induced pain on head, a randomized controlled clinical trial between sham and experimental group were designed applying on GV20 with four EX-HN1 via wearable LLLAT device emitting five acupoints for 30 min simultaneously.

**Table 1:**
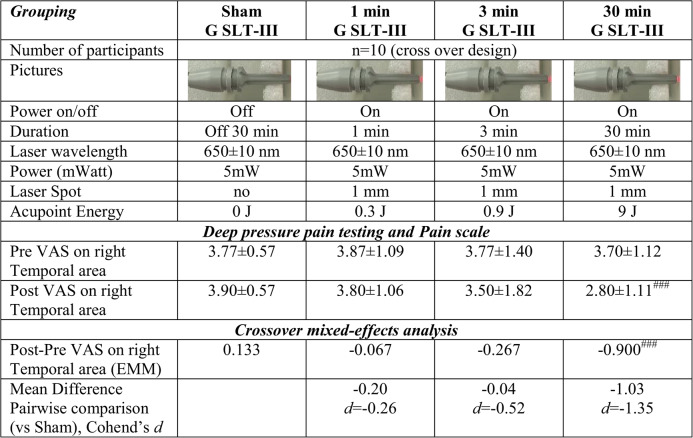
Preliminary effectiveness of LLLAT for 1 min, 3 min, and 30 min on LI4 acupoint

*LLLAT,* Low-level Laser Acupoint Therapy; *LI4, *Hegu acupoint; *VAS,* Visual Analog Scale score. Data of Pre VAS and Post VAS show mean±standard deviation. Post-Pre VAS show Estimated Marginal Means (EMM), Mean Difference Pairwise comparison (vs Sham) and Cohend’s *d *were analyzed by crossover mixed-effects analysis. ^###^*p* < 0.001 significantly different between Sham (Off) and Intervention

## Method

### Participant

Forty-eight healthy volunteers aged between 20 and 55 years and signed Informed Written Consent to participate in deep pressure threshold testing with or without wearable Low-level Laser Acupoint Therapy (LLLAT). None of the subjects were on any regular medication. The trial was performed according to the principles of the Declaration of Helsinki and the protocol was approved by the Research Ethical Committee of China Medical University Hospital, Taiwan (CMUH110-REC1-065) approved period from 2021.06.07 to 2025.06.06.

### Deep pressure pain testing on head and pain scale

Touch Test ® Sensory Evaluator (Semmes-Weinstein Monofilaments) Product Number NC12775-20, Evaluator size: 6.65, target force 300 g to generate deep pressure pain on individual right temporal head and Visual Analog Scale (VAS) score (0 [no pain] to 10 [worst pain ever]) on individual right temporal area over different test sites. All pre- and post-pain VAS scales were determined with three stimulus repetitions of 300 g force, the mean of three times which was taken as the final value.

### Low-level laser acupoint therapy (LLLAT)

All Low-level Laser Acupoint projected laser wavelength 650 ± 10 nm and laser power 5mW. In the preliminary study, the Low-level Laser Acupoint Therapies, a cFDA proved class II medical device, semiconductor Laser Therapeutic Instrument (G SLT-III, Shenzhen Tianjiquan Health Science &Technology Group Co., Ltd., Guangdong, China). In current primary study, a wearable semiconductor Laser Therapeutic Instrument (MBU-LA01, MiraBrain Technology (Zhuhai), Guangdong, China) targeting GV20 and four EX-HN1 acupoints was used as LLLAT. The five laser acupoint projections of wearable LLLAT were projected at the same time for 30 min.

### Protocol of preliminary study for LLLAT duration

Effectiveness of self-manual LLLAT projecting 650 ± 10 nm Laser with 5 mW for sham (off), 1 min, 3 min, and 30 min on left LI4 acupoint was investigated from ten voluntary participants in a repeated-measures crossover design. Before LLLAT on LI4, all participants underwent Deep pressure pain testing via 300 g monofilament and assess Pain VAS scale determined with three stimulus repetitions of 300 g force. In each week, ten participants were cross-overly assigned to sham LLLAT on LI4 (no power, 30 min), LLLAT on LI4 for 1 min, LLLAT on LI4 for 3 min, or LLLAT on LI4 for 30 min. After LLLAT on LI4, all participants underwent Deep pressure pain testing via 300 g monofilament and assess Pain VAS scale determined with three stimulus repetitions of 300 g force. The effectiveness of Low-level Laser Acupoint Therapies projecting 650 ± 10 nm Laser with 5 mW for sham (off), 1 min, 3 min, and 30 min on left LI4 acupoint were determined by crossover mixed-effects analysis. Based on the findings from the preliminary study, LLLAT of GV20 in the primary study was applied for 30 min.

### Primary study

Effectiveness of wearable Low-level Laser Acupoint Therapy on GV20 with four EX-HN1 acupoints was investigated in forty-eight participants randomized assigned (computer-based randomization) into either wearable LLLAT group (*n* = 24) or sham group (*n* = 24). This is a prospective, triple-blinded trial, in which participants could not tell the differences between sham and power-on as well as the follow-up assessors and the statistical analyzers were both blinded in the wearable LLLAT group on GV20 with four EX-HN1 acupoints or sham group.

### Protocol

**Fig. 1:**
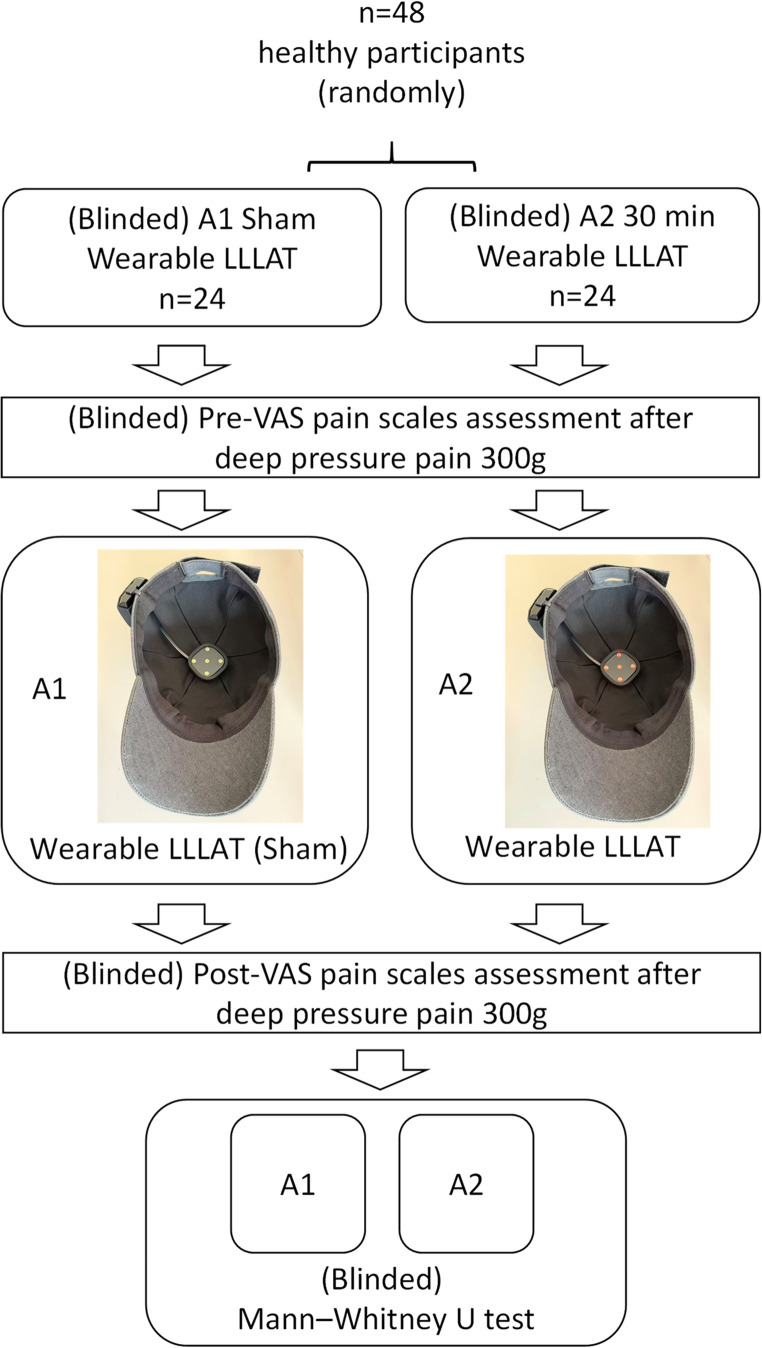
Protocol of effectiveness of wearable low-level laser acupoint therapy on GV20 with four EX-HN1 acupoints. LLLAT: Low-level Laser Acupoint Therapy; VAS: Visual Analog Scale; A1, A2: Blinded Grouping; A1: LLLAT Sham group applying on GV20 and four EX-HN1 acupoints; A2: LLLAT applying on GV20 and four EX-HN1 acupoints

The protocol was shown in Fig. 1. Forty-eight participants randomized blindedly assigned (computer-based randomization) into either wearable LLLAT group (A1, *n* = 24) or sham group (A2, *n* = 24) after signing Informed Written Consent. Before LLLAT, all participants underwent Deep pressure pain testing via 300 g monofilament and assess Pain VAS scale determined with three stimulus repetitions of 300 g force. In A1 group, 24 participants were assigned to wearable sham LLLAT (no power, 30 min) on Baihui (GV20) with four Sishencong (EX-HN1). In A2 group, 24 participants were assigned to wearable LLLAT (650 nm, 5mW, 30 min, about 9 J each acupoint) on Baihui (GV20) with four Sishencong (EX-HN1). After LLLAT, all participants underwent Deep pressure pain testing via 300 g monofilament and assess Pain VAS scale determined with three stimulus repetitions of 300 g force. All data from groups A1 and A2 were coded and submitted to an independent statistician for statistical analyses, with group allocation (LLLAT or sham) remaining blinded to ensure unbiased evaluation (Fig. 1).

### Statistical analysis

In the preliminary study of repeated measurements crossover design, Post-Pre VAS after self-manual Low-level Laser IL4 Acupoint Therapy comparing among sham, therapeutic duration 1 min, therapeutic duration 3 min, and therapeutic duration 30 min intervention were analyzed by crossover mixed-effects analysis. All the VAS pain scales (each VAS pain scale was averaged from three times deep pressure pain testing) after GV20-based Wearable Low-level Laser Acupoint Therapy were analyzed in all participants who were randomly assigned A1 and A2. Normality of the data was assessed using the Shapiro–Wilk test, which indicated that at least one group deviated from a normal distribution. Therefore, a non-parametric approach was adopted. Differences between Group A1 and Group A2 were evaluated using the Mann–Whitney U test. The significant level used for the statistical analysis with two-sided testing was 5%; therefore, *P* < 0.05 indicated significance. Data values were presented by mean ± standard deviation. All analyses were performed using the PASW Statistics (SPSS) software program (version 12) for Windows.

**Table 2:** Effectiveness of wearable LLLAT on GV20 and four EX-NH 1 acupoints via MBU-LA01

*Grouping*	Sham wearable	30 min wearable
	MBU-LA01	MBU-LA01
Number of participants	n=24	n=24
Power on/off; duration	Off;	On;
Duration	Sham 30 min	On 30 min
Laser wavelength	650±10 nm	650±10 nm
Watt	5mW	5mW
Laser Spot; number of Spot	No	1 mm x5
Acupoint Energy	0 J	9J x 5
*Deep pressure pain testing and Pain scale*		
Pre VAS on right Temporal area	4.47±0.97	4.50±1.13 ^NS^
Post VAS on right Temporal area	4.40±1.32	3.00±1.26***
Post-Pre VAS on right Temporal area	0.03±0.93	-1.40±1.38***

*LLLAT,* Low-level Laser Acupoint Therapy. GV20: Baihui. EX-HN1: Sishencong. *VAS,* Visual Analog Scale score. Data show mean±SD. ^NS^ Non-significantly different between Sham MBU-LA01 and MBU-LA01. ***p < 0.001 very highly significantly different between Sham MBU-LA01 and MBU-LA01 using the Mann–Whitney U test

## Results

The preliminary effectiveness of laser energy output for sham (off), 1 min, 3 min, 30 min and effectiveness of pain reduction of LLLAT manually applied on LI4 acupoint via a Semiconductor Laser Therapeutic Instruments G SLT-III was shown in Table 1.

In a crossover mixed-effects analysis (*n* = 10), the intervention of 30 min G SLT-III demonstrated a large and statistically significant effect versus sham (mean difference=-1.03, 95% CI [-1.63, -0.43], *p* = 0.001, Cohen’s *d* = 1.35). Comparing with pre-post-intervention, the post VAS pain scores on right Temporal area were similar with pre VAS score in sham group, 1 min G SLT-III group and in 3 min G SLT-III group. (Table 1).

Effectiveness of wearable LLLAT on GV20 and four EX-NH1 acupoints via MBU-LA01 was shown in Table 2. Pre VAS pain scales on right Temporal area in sham group of wearable MBU-LA01 from 24 participants were non-significantly (U = 303.5, Z = 0.32, *p* = 0.755, with a negligible effect size (*r* = 0.046) similar with those in wearable MBU-LA01 from 24 participants (4.47 ± 0.97 vs. 4.50 ± 1.13). The Hodges-Lehmann estimate indicated a median difference near 0, with a 95% confidence interval of [-2, 2]. Post VAS pain scales (3.00 ± 1.26) in wearable MBU-LA01 from 24 participants (three times per participant) were very highly significantly (U = 464.0, Z = 3.63, *p* = 0.00028 < 0.0001, with a large effect size (*r* = 0.52) lower than those in sham group of wearable MBU-LA01 from 24 participants (4.40 ± 1.32). The Hodges-Lehmann estimate indicated a median difference of -1.33, with a 95% confidence interval of [-5.00, 2.00]. Post VAS minus Pre VAS on right Temporal area in wearable MBU-LA01 group were very highly significantly (U = 491.5, Z=-4.2, *p* = 0.000026 < 0.001 with a large effect size (*r* = 0.53) lower than those in sham group of wearable MBU-LA01 (-1.40 ± 1.38 vs. 0.03 ± 0.93) demonstrated a statistically significant difference between the two groups. The Hodges-Lehmann estimate indicated a median difference of -1.33, with a 95% confidence interval of [-1.67, -0.50].

### Safety of LLLAT

No adverse events, including burns, skin changes, nausea, self-discomfort or dizziness, were reported from all healthy participants during and after applying wearable Low-level Laser Acupoint Therapy (LLLAT) emitting laser wavelength 650 ± 10 nm and laser power 5 mW for 30 min via the MBU-LA01 Semiconductor Laser Therapeutic Instrument.

## Discussion

Our major findings can summarized: Deep pressure pain induced by evaluator size 6.65 for targeting force 300 g on individual right temporal head after wearable Low-level Laser Acupoint Therapy on Baihui (GV20) with four Sishencong (EX-HN1) were significantly reduced comparing with the sham group via MBU-LA01 Semiconductor Laser Therapeutic Instrument emitting five laser acupoint laser wavelength 650 ± 10 nm and laser power 5 mW simultaneously for 30 min. No adverse events, including burns, skin changes, nausea, self-discomfort or dizziness, were reported but effective pain reduction was reported after applying wearable Low-level Laser Acupoint Therapy, suggesting wearable design of multiple LLLAT at the same time (laser wavelength 650 ± 10 nm and laser power 5 mW for 30 min) can be safely and user-friendly applied.

In the preliminary study “Does 1 min, 3 min, or 30 min Low-level Laser Acupoint Therapy for Pain on Head”, given the crossover repeated-measures design, the sample size of 10 subjects provides adequate power (> 80%) to detect large within-subject effects (Cohen’s d > 0.8), assuming moderate to high within-subject correlation. In the main study “Effectiveness of GV20-based Wearable Low-level Laser Acupoint Therapy for Pain on Head”, a post hoc power analysis indicated that with a sample size of 24 participants per group and a large observed effect size (*r* = 0.52), the study achieved an estimated statistical power of approximately 0.97–0.99 (α = 0.05, two-tailed), suggesting that the sample size was sufficient to detect the observed effect.

Low-level laser Therapy (LLLT) including Low-level Laser Acupoint Therapy (LLLAT), triggers a chemical reaction in cells and/or in acupoint called photobiomodulation, helping reducing pain [13]. Low-level Laser Acupoint Therapy (LLLAT) is expected to produce a synergistic therapeutic effect by combining the acupoint stimulation with the photobiomodulatory effects of laser therapy. Moreover, because the laser is emitted directly from the handle tip generating pressure force into the acupoint, it minimizes energy loss through the skin layers and enables the delivery of a sufficient amount of energy precisely to the targeted acupoints.

In the current study, GV20-based wearable Low-level Laser Acupoint Therapy using Laser wavelength 650 ± 10 nm and laser power 5 mW for 30 min were significantly reduced deep pressure right temporal head pain. GV20 was often suggested an effective treatment for chronic tension-type head pain [14] and migraine [9]. The therapeutic mechanism of low-level laser acupoint therapy on head pain are not fully elucidated and current evidence suggests that might relate to inhibiting descending pain modulatory system, up-regulating endogenous opioid peptides [15], exerting anti-inflammatory effects through NOX-ROS-NLRP3 pathway [16], regulating neurotransmitters release as well as improving central pain sensitization [17]. Furthermore, the 650 nm wavelength specifically modulate neural transmission by regulating acetylcholinesterase, substance P, leu-enkephalin, and c-Fos/GFAP expression [18], which might support its potential efficacy in pain relief.

According to a data mining study from randomized controlled clinical trials, the most frequently used points to treat migraine were GB20, LR3, GV20, Taiyang, LI4 and TE5 [9]. In the current study, the IL4 is the easiest acupoint for-administered manual LLLAT. In contrast, GV20 is the most suitable acupoint for wearable LLLAT, as it is easily accessible on the top of head. In previous study, LLLAT was effective in treating chronic migraine for reducing patients’ pain via emitting energy of 4.5 J each acupoint by each point (810 nm, 150 mW, 30 s, LaserPen-Expert 511 A, Reimers & Janssen GmbH, Germany) including Hegu (LI4).

### Sufficient acupoint LLLAT energy

One randomized clinical trial provided clinical evidence for the safety and efficacy of 650 nm LLLAT in managing of non-specific chronic low back pain [18]. Laser wavelength 650 nm and laser power 5mW for 30 min of LLLAT could reduce deep pressure pain induced by evaluator size 6.65 for targeting force 300 g. Based on E (energy, J) = P (power, W)x t (time, sec), some studies apply LLLAT (650 nm and laser power 20 mW for 3 min per acupoint) about 3.6 J each acupoint [19], whereas our study apply LLLAT (650 nm and laser power 5 mW for 30 min per acupoint) about 9 J each acupoint. In sum, total energy of LLLAT emitting 650 nm and laser power 5mW for 30 min per acupoint is 2.5 times greater than LLLAT emitting 650 nm and laser power 20 mW for 3 min per acupoint. We suggest that wearable LLLAT emitting 650 nm and laser power 5mW for 30 min per acupoint is safe, efficient and effective for multiple acupoint stimulation.

### Limitation

Deep pressure pain on Head in healthy participant can only tell pain sensation on head but cannot represent headache, tension-induced headache, or migraine. Therefore, future studies will be required to answer the effectiveness of GV20-based Wearable Low-level Laser Acupoint Therapy for clinical headache such as tension-induced headache or migraine. Besides, different head size from adult and child might impact the precise acupoint location and, if targeting other acupoints, other style wearable Low-level Laser Acupoint Therapies should be designed to target different acupoints for treating pain.

## Conclusion

This study suggested that Low-level Laser Acupoint Therapy 650 nm 5 mW for 30 min emitting on GV20 and four EX-HN1 acupoints simultaneously could effectively reduce deep pressure pain on temporal head.

## Data Availability

All statistical data is presented in the manuscript. Origin data in each test will be provided upon special request to the corresponding author.
